# Constructing Selenium Nanoparticles with Enhanced Storage Stability and Antioxidant Activities via Conformational Transition of Curdlan

**DOI:** 10.3390/foods12030563

**Published:** 2023-01-27

**Authors:** Xindong Xu, Yuxue Pan, Xiaoying Liu, Zhong Han, Shan Chen

**Affiliations:** 1 College of Food Science and Engineering, South China University of Technology, Guangzhou 510641, China; 2 College of Light Industry and Food Engineering, Guangxi University, Nanning 530004, China; 3 Guangdong Provincial Key Laboratory of Intelligent Food Manufacturing, Foshan University, Foshan 528225, China; 4 Collaborative Innovation Center for Guangxi Sugar Industry, Guangxi University, Nanning 530004, China

**Keywords:** selenium nanoparticle, curdlan, storage stability, conformational transition, triple helix

## Abstract

Selenium nanoparticles (SeNPs) are among the emerging selenium supplements because of their high bioactivity and low toxicity. However, bare SeNPs are prone to activity loss caused by aggregation and sedimentation. This study aims to stabilize SeNPs with curdlan (CUR), a polysaccharide, to maintain or even enhance their biological activity. Herein, the stable SeNPs were constructed via the unique conformational transition of CUR induced by alkali-neutralization (AN) pretreatment. The physicochemical properties and structures of the prepared SeNPs were characterized by dynamic light scattering (DLS), UV-visible spectroscopy, Fourier-transform infrared spectroscopy (FTIR), X-ray diffraction (XRD), transmission electron microscopy (TEM), and free-radical-scavenging activity assays. The results show that most SeNPs are stabilized within the triple helix of CUR that has been pretreated with high-intensity AN treatment. These amorphous, small-sized (average size was 53.6 ± 17.7 nm), and stabilized SeNPs have significantly enhanced free-radical-scavenging ability compared to the control and can be well-stabilized for at least 240 days at 4 °C. This work indicates that CUR, as a food additive, can be used to well-stabilize SeNPs by AN pretreatment and provides a facile method to prepare and enhance the stability and bioactivity of SeNPs via triple-helix conformational transition.

## 1. Introduction

Selenium plays a key role in regulating immunity and sperm activity [1], and selenium levels in the body can affect the occurrence of inflammation and diseases [2]. A recent analysis on the relationship between COVID-19 cases and the regional selenium status of China has revealed that the pathogenicity and lethality of COVID-19 are higher in regions with low selenium levels (except for Wuhan), indicating the potential role of selenium in the prevention and treatment of viral infections [3]. Among numerous selenium supplements, selenium nanoparticles (SeNPs) reportedly have lower toxicity and higher biological activity than organic selenium (e.g., selenomethionine and Se-methyl selenocysteine) and inorganic selenium (e.g., sodium selenite (Na_2_SeO_3_)) [4,5,6,7]. SeNPs can be synthesized chemically [8] or by using physical procedures [9] or can be obtained by biological ways [10]. The main method of preparing SeNPs is the chemical reduction in selenium salts, such as Na_2_SeO_3_ and sodium selenosulfate [11,12,13,14]. Synthesis of SeNPs by redox reaction of Na_2_SeO_3_ and ascorbic acid is a relatively simple method. The reduction in selenite with ascorbic acid can be described by the general reaction as follows [15].

$$H_{j}{\mathrm{SeO}}_{3}^{j-2}+2C_{6}H_{8-i}O_{6}^{-i}+\left(2\left(i+1\right)-j\right)H^{+}↔{\mathrm{Se}}^{0}+2C_{6}H_{6}O_{6}+3H_{2}O$$

where the possible hydrolyzed species are defined by *j* = 0–2 and *i* = 0–2. However, SeNPs are prone to aggregate into large particles without stabilizers, which can seriously affect their bioavailability and bioactivity [16]. Therefore, it is necessary to use suitable stabilizers to stabilize SeNPs.

Natural polysaccharides are increasingly being used as stabilizers for SeNPs because of their excellent biocompatibility and the large number of hydroxyl groups [17]. Chitosan, dextran, arabinogalactans, and carrageenan, as well as nonthermal techniques such as ultrasound, have been used to stabilize SeNPs, and the enhanced bioactivities of these SeNP composites have been confirmed [18,19,20,21,22]. Polysaccharides with a unique triple-helix conformation have also been used to stabilize SeNPs [23,24]. Triple-helix polysaccharides (THPs) also have a large number of hydroxyl groups, and their triple-helix conformation enables them to undergo self-assembly under certain conditions [25,26,27,28]. The coupling of this self-assembly process with SeNP synthesis provides a new idea for a better stabilization and bioactivities of SeNPs because SeNPs have been reported to be encapsulated within the helical cavity of THPs such as *Auricularia* polysaccharide and lentinan [24,29]. However, some THPs represented by lentinan have low extraction rates and other problems [30], which may limit their large-scale application in the construction of THP-SeNP composites. It is necessary to find a reliable source of low-cost THP instead of relying on extracted THP.

Curdlan (CUR) is a linear *β*-(1,3) glucan without branched chains [31,32], which is extensively used in the food industry as a safe food additive permitted by the United States FDA [33]. CUR is usually produced by the fermentation of bacteria and it is easy and cheap to purchase from many biotechnology companies [32]. Hence, it is appropriate and worthwhile to stabilize SeNPs with CUR instead of other THPs. To the best of our knowledge, though an article has reported on SeNPs stabilized with carboxylic CUR [11], the safety of carboxylic CUR is still a concern. The purpose of carboxylation modification is to enhance the water solubility of CUR. However, our recent work has demonstrated that alkali-neutralization (AN) treatment can effectively improve the water solubility of CUR by inducing the dissociation of triple-helix aggregates (THAs) [34]. In brief, CUR powder was first dissolved in a sodium hydroxide aqueous solution because of the denaturation of the triple helices; then, the mixture was neutralized by hydrochloric acid to renature the triple helices. We believe that this pretreatment is simpler and safer for fabricating the CUR-SeNP composites.

According to our previous confirmation, CUR has different self-assembly behaviors at different AN concentrations [34]. Under a low-intensity AN treatment (*C*_NaOH/HCl_ = 0.1 mol/L), the THAs first disaggregate into small-sized THAs and independent triple helices (ITHs) after alkali treatment; then, these ITHs re-aggregate to THAs after the neutralization treatment. Under a high-intensity AN treatment (*C*_NaOH/HCl_ = 1.0 mol/L), THAs first disaggregate completely into random coils after alkali treatment; then, these random coils renature to ITHs, but few ITHs further aggregate to THAs. The difference between the two self-assembly behaviors of CUR lies in the presence or absence of unwinding-rewinding of ITHs. From this, it can be hypothesized that different self-assembly behaviors of CUR may affect the structure and physicochemical properties of CUR-SeNP composites because the SeNPs may be encapsulated inside the triple helix through the renaturation of ITHs. Therefore, there is a need to verify how this difference significantly affects (if there is any effect) the physicochemical properties of CUR-SeNP composites.

This study aims to stabilize SeNPs using unmodified CUR and to resolve the effects of CUR with different intensities of AN pretreatment on the stabilization of SeNPs. We found that high-intensity AN-pretreated CUR was more suitable for stabilizing SeNPs, which have good storage stability and antioxidant activity. This work would help to promote the use of CUR, a food additive, in the fabrication of selenium supplements that are of interest in the field of food nutrition and functional foods.

## 2. Materials and Methods

### 2.1. Materials

Curdlan (M*_w_* = 1.55 × 10^4^ Da) was purchased from Yuanye Bio-Technology Co., Ltd. (Shanghai, China) and used without any additional purification [34]. The dialysis bag (M*_w_* cutoff = 3500 Da) was purchased from Solarbio Science & Technology Co., Ltd. (Beijing, China). Sodium selenite (Na_2_SeO_3_) and ascorbic acid were purchased from Sigma–Aldrich Chemical Co., Ltd. (St. Louis, MO, USA). All chemicals and solvents were of analytical grade, and ultrapure water was used in all experiments.

### 2.2. Alkali-Neutralization Pretreatment of CUR

Alkali-neutralization (AN) pretreatment was performed according to our previous work with slight modifications [34]. In brief, 0.6 g of CUR powder was first slowly added into the 30 mL of ultrapure water under stirring magnetically for 12 h at 25 °C; then, the aqueous suspension of CUR was filtered and the filtrate was collected for subsequent steps. The filtrate was divided into two equal portions, and the NaOH solutions were separately added to make the final NaOH concentrations 0.1 and 1.0 mol/L. The mixture was stirred magnetically at 25 °C for 6 h to induce the denaturation of CUR. Finally, the curdlan dispersion was neutralized (pH 7) and immediately used for the preparation of CUR-SeNP composites.

### 2.3. Preparation of CUR-SeNPs

CUR-SeNPs were prepared according to the following procedure. The reaction of sodium selenite with ascorbic acid was carried out as described by Li, Li, Wong, Chen, Zhang, Liu, and Zheng [35] with slight modifications. In brief, the CUR solutions with different intensities of AN pretreatment were immediately separately mixed with 250 μL of 0.1 mol/L Na_2_SeO_3_ aqueous solution, and magnetically stirred for 1 min. Then, 500 μL of 0.2 mol/L ascorbic acid aqueous solution was separately added into the mixtures to start the synthesis reaction of SeNPs, and the resulting mixtures were stirred for 24 h in the dark at 25 °C. At the end of the reaction, the mixtures were dialyzed against ultrapure water for 48 h, and the water was changed every 12 h. After the necessary analysis of the dialyzed sample, it was freeze dried with a SCIENTZ 18N (Xinzhi, China) freeze dryer and stored at 4 °C and 25 °C. The samples were named CUR-SeNPs-L and CUR-SeNPs-H according to the intensity of AN pretreatment. Control sample without CUR was prepared as above and named SeNP control. Control samples without SeNPs were prepared as described previously and named CUR-L and CUR-H according to the intensity of AN pretreatment.

### 2.4. Hydrodynamic Diameter Distribution and Zeta Potential Measurements

The hydrodynamic-diameter distribution and zeta potential of the CUR-SeNP composites were measured by dynamic light scattering (DLS) with a ZETASIZER NANO ZS90 (Malvern Panalytical, Malvern, UK) at 25 °C [36]. The diluted sample solution was injected into the cuvette along the inner wall with a pipette, and the liquid surface height did not exceed the maximum height specified by the instrument, and then the cuvette was placed into the test port and covered with the cuvette, and the test chamber cover was closed for measurement. A PCS1115 cuvette was selected for the hydrodynamic-diameter distribution measurement. The refractive and absorption indices were set at 2.92 and 0.30, respectively. The equalization time of all samples was set as 90 s. A DTS1070 cuvette was selected for zeta potential measurement, and the number of zeta runs was set to 12.

### 2.5. UV-vis Absorption Spectroscopy

About 2 mL of the diluted sample was drawn into a quartz cuvette and transferred into a corrected UV-2501(PC)S (Shimadzu, Tokyo, Japan) UV-vis absorption spectrometer. The scanning wavelength range was 200–800 nm with a resolution of 1 nm [36].

### 2.6. Fourier-Transform Infrared (FTIR) Spectroscopy

The KBr-disk method was used for FTIR analysis [18]. Briefly, the lyophilized sample was suitably ground to powder and mixed with dried KBr powder for grinding and drying. Then, the mixture was transferred into the mold for a 3 min hydraulic treatment to obtain a test piece, which was immediately tested in a TENSOR II (Bruker, Billerica, MA, USA) FTIR spectrometer. The FTIR spectra of the samples were recorded in the scanning wavenumber range of 4000–400 cm^−1^ and at a resolution of 0.5 cm^−1^. Each sample was scanned 16 times. The results were analyzed by an OPUS 8.5 (Bruker, Billerica, MA, USA).

### 2.7. X-ray Diffraction (XRD)

The lyophilized sample was suitably ground to powder and measured by a D/MAX 2500 V (Rigaku, Tokyo, Japan) X-ray diffractometer using Bragg–Brentano (θ, 2θ) geometry and Cu-Kα radiation. The operational parameters were as follows: scanning range of 5–80°; scanning speed of 4°/min; and the voltage and current were 40 kV and 40 mV, respectively [27]. The results were analyzed using a Jade 5.0 (MDI, Livermore, CA, USA).

### 2.8. Transmission Electron Microscopy (TEM)

The samples were observed with a HT-7700 (Hitachi, Tokyo, Japan) transmission electron microscope [37]. In a typical procedure, before the observation, the sample was dripped onto a copper grid using a syringe and air dried at room temperature in an ultra-clean bench. Accelerating voltage set to 100 kV. Mean particle size of SeNPs in electron microscopy photographs was calculated by Nano Measurer 1.2.5 software (Xujie, Shanghai, China).

### 2.9. Assay of Reactivity of CUR-SeNP Composites with Free Radicals

#### 2.9.1. Hydroxyl-Radical-Scavenging Activity Assay

Hydroxyl-radical-scavenging activity was determined by the procedure described by Mao, Zou, Feng, Wang, Zhao, Ye, Zhu, Wu, Yang, and Wu [38] with slight modifications. In a typical procedure, 1.0 mL of 9.0 mmol/L FeSO_4_ aqueous solution, 1.0 mL of 9.0 mmol/L salicylic acid ethanol solution, and 1.0 mL of sample were mixed. After adding 1.0 mL of 9.0 mmol/L H_2_O_2_, the mixture was incubated for 30 min at 37 °C. The absorbance was measured at 510 nm. The hydroxyl-radical-scavenging ability was calculated using the following equation:

Hydroxyl-radical-scavenging ability (%) = [1 − (A_1_ − A_2_)/A_0_] × 100 (1)

where A_1_, A_2_, and A_0_ represent the absorbance in the presence of sample, the absorbance in the absence of H_2_O_2_ (water instead), and the absorbance in the absence of sample (water instead), respectively.

#### 2.9.2. Superoxide-Radical-Scavenging Activity Assay

Superoxide-radical-scavenging activity was determined by the procedure described by Li [39] with slight modifications. In a typical procedure, 50 μL of 60 mmol/L pyrogallol solution (1 mmol/L HCl, 37 °C) was mixed with 2900 μL of Tris-HCl buffer (0.05 mol/L, pH 7.4, 37 °C) containing 1 mmol/L Na_2_EDTA. The absorbance was measured at 325 nm every 30 s for 10 min. The superoxide-radical-scavenging ability was calculated using the following equation:

Superoxide-radical-scavenging ability (%) = (ΔA_0_ − ΔA_1_)/ΔA_0_ × 100 (2)

where ΔA_0_ represents the increment in A_325nm_ of the mixture without a sample, and ΔA_1_ represents the increment in A_325nm_ of the mixture with a sample.

### 2.10. Statistical Data Analysis

All determinations were performed in duplicate, and the results are presented as the mean ± standard deviation. Statistical analysis was conducted using IBM SPSS 20.0 software. An analysis of variance (ANOVA) using Turkey’s multiple-range test was performed for mean comparison at *p* < 0.05.

## 3. Results and Discussion

### 3.1. Hydrodynamic-Diameter Distribution and Zeta Potential of CUR-SeNPs

**Figure 1:**
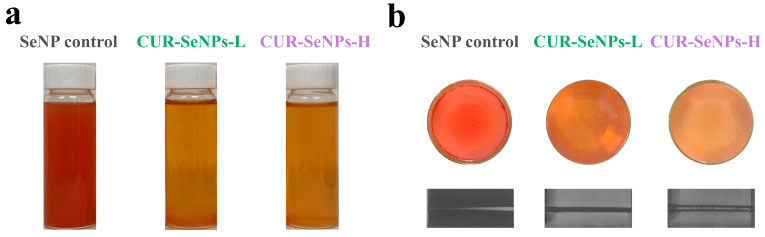
(**a**) Front view, (**b**) bottom view and grayscale photograph under 650 nm-laser light of CUR-SeNPs samples.

**Figure 2:**
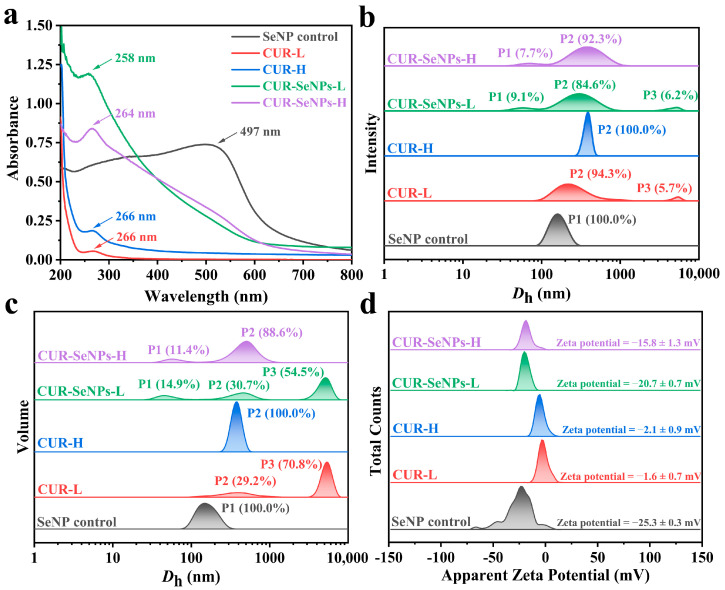
(**a**) The ultraviolet spectra, (**b**) hydrodynamic-diameter distribution by intensity, (**c**) hydrodynamic-diameter distribution by volume, and (**d**) zeta potential of CUR-SeNP samples.

Generally, at a given selenium concentration, the SeNPs with smaller particle size exhibit a more yellowish and more transparent appearance [20,24,36]. As shown in Figure 1a, both CUR-SeNPs were more yellowish in color and more transparent than the SeNP control, indicating that the SeNPs in both composites were stabilized and had a smaller particle size. The bottom photos of the samples were also obtained (Figure 1b). It was clear that dark red particles appeared at the bottom of the SeNP control, indicating that slight aggregation and sedimentation of SeNPs had occurred. In addition, we found a few translucent, small, and colored particles at the bottom of the CUR-SeNPs-L sample, which may be due to re-formation of triple-helix aggregates (THAs) entrapping some SeNPs under the low-intensity AN treatment. Three samples were also irradiated with a 650 nm laser to determine the light transmission (Figure 1b), and all of them showed optical pathways. According to the Tyndall phenomenon, it indicated to some extent that the average particle size of all three samples was less than 650 nm. As shown in Figure 2a, the maximum absorption wavelengths (λ_max_) of the three samples were 497, 258, and 264 nm, respectively, and their absorbances started to increase significantly from 600 nm, which was consistent with the typical UV-vis spectroscopic pattern of SeNPs [40]. The introduction of SeNPs seems to change the UV absorption of CUR, which is caused by the superposition of the absorption spectra of CUR and SeNPs. As shown in Figure 2a, CUR-L and CUR-H have the same λ_max_ (266 nm), although they are very different in conformation [34]. However, the particle sizes of SeNPs in CUR-SeNPs-L (45.4 ± 21.1 nm) and CUR-SeNPs-H (53.6 ± 17.7 nm) were different, as supported by our subsequent TEM results. Based on a previous study on the relationship between the particle size of SeNPs and their absorption spectral characteristics [40], the SeNPs with larger particle size have the larger λ_max_. For example, the λ_max_ of SeNPs with an average particle size of 70.9 ± 9.1 nm is approximately 270 nm, while the λ_max_ of SeNPs with an average particle size of 101.6 ± 9.8 nm is approximately 300 nm. Based on this relationship, it is reasonable to assume that the λ_max_ of SeNPs in both CUR-SeNPs-L and CUR-SeNPs-H should be less than 266 nm, and the λ_max_ of the former is smaller. Therefore, the larger SeNPs in CUR-SeNPs-H resulted in a weaker blueshift (266 nm to 264 nm) in λ_max_, while CUR-SeNPs-L had a stronger blueshift (266 nm to 258 nm).

To further define the particle size of CUR-SeNP samples, the hydrodynamic diameter (*D*_h_) distributions of the samples were measured, and the results are shown in Figure 2b,c and Table 1. As shown in Table 1, the z-average size of the SeNP control, CUR-SeNPs-L, and CUR-SeNPs-H was 159.1, 230.1, and 280.6 nm, respectively, indicating that CUR increased the overall *D*_h_. As shown in Figure 2b,c (original graphs can be found in Figure S1), the *D*_h_ distribution by intensity and volume demonstrated this pattern with increased clarity. The P3 peaks with an average *D*_h_ of 4737 nm and 4978 nm (by intensity) in CUR-SeNPs-L and CUR-L confirmed the presence of THAs in the solution. The size of THAs was reported to be in the micrometer scale [41]. In CUR-SeNPs-L and CUR-SeNPs-H, P1 peaks could be observed, which were not present in their control samples without SeNPs, indicating that the P1 peaks were generated by free SeNPs. Therefore, after confirming the reasons for the formation of P1 peak and P3 peak in the *D*_h_ distribution, it is reasonable that the P2 peak was attributed by independent triple helices (ITHs) dissociated from THAs. Furthermore, as shown in Table 1, the average *D*_h-P2_ (both by intensity and volume) of both CUR-SeNP samples was higher than that of their control samples without SeNPs, indicating that SeNPs were bound to ITHs. However, the binding between SeNPs and CUR was different in the two CUR-SeNP samples. As we described in the introduction, CUR has different conformational transitions induced by different intensities of AN pretreatment. In the formation of CUR-SeNPs-L, SeNPs cannot be wrapped inside the ITHs through the reaggregation of ITHs to THAs, so SeNPs can only be attached to the outside of the ITHs or be trapped inside the re-aggregated THAs [34]. Comparatively, in the formation of CUR-SeNPs-H, the ITHs could be reformed by the rewinding of random coils, allowing more SeNPs to be stabilized inside the triple helix by chemical interaction and physical encapsulation during the rewinding. This conclusion was confirmed by the larger *D*_h-P2_ (both by intensity and volume) of CUR-SeNPs-H compared to CUR-SeNPs-L.

Moreover, the average zeta potentials (pH 7, 25 °C) of SeNP control, CUR-SeNPs-L, and CUR-SeNPs-H were measured at −25.3, −20.7, and −15.8 mV, respectively (Figure 2d, and original graph can be found in Figure S2). The zeta potential difference between CUR-SeNPs-L and CUR-SeNPs-H also confirmed that the SeNPs in CUR-SeNPs-H were encapsulated inside the triple helices, because the SeNPs were entrapped in the cavities of the triple helix, shielding the partial negative charges of SeNPs and leading to smaller negative potentials.

### 3.2. Molecular Structure and Crystalline Structure of CUR-SeNPs

**Figure 3:**
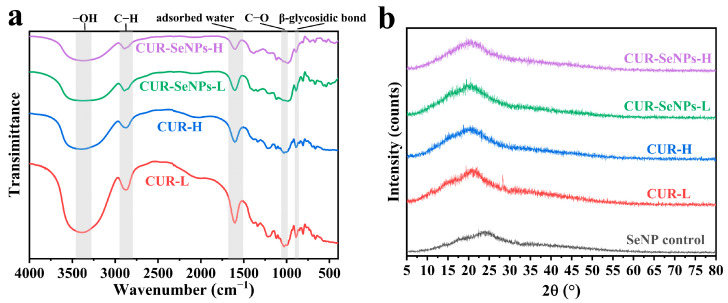
(**a**) The FTIR spectra and (**b**) XRD patterns of CUR-SeNP samples.

FTIR and XRD are effective methods for analyzing the structural characteristics of CUR-SeNPs. Figure 3a shows the FTIR spectra of CUR-L, CUR-H, CUR-SeNPs-L, and CUR-SeNPs-H, respectively. In the case of CUR-L, the stretching or vibrations corresponding to the main peaks were as follows: 3385 cm^−1^ (stretching vibration of –OH), 2881 cm^−1^ (stretching vibration of C–H), 1606 cm^−1^ (absorption peak of the adsorbed water blending vibration), 1002 cm^−1^ (stretching vibration of C–O), and 890 cm^−1^ (β-glycosidic linkage) [34]. The differences in the absorption peaks of these stretching vibrations between the samples are detailed in Table 2.

There is a characteristic absorption peak of the β-conformation at 890 cm^−1^ in all spectra, indicating the presence of curdlan in all samples. The –OH stretching vibration peaks of both CUR-SeNP samples were shifted to the lower wavenumbers (3379 cm^−1^ and 3376 cm^−1^) compared to that of AN-pretreated CURs (3385 cm^−1^), indicating that SeNPs were stabilized by the interaction between Se atoms and hydroxyl groups, and this result was similar to that of the other THP-SeNP composites [23,24]. From the electronegativity of H (χ = 2.20), O (χ = 3.44), and Se (χ = 2.55) atoms, it can be inferred that the strength of the H···Se hydrogen bonding interaction is lower than the intra- or intermolecular H···O hydrogen bonding of the CUR, which was confirmed by a recent molecular simulation study that reported the important role of H···Se hydrogen bonding in the formation of functional materials [42]. Therefore, we attributed the red shift of the –OH stretching vibration peak to Se···H hydrogen bonding. Furthermore, the larger wavenumber shift of CUR-SeNPs-H suggested that more SeNPs were stabilized compared to CUR-SeNPs-L, which was consistent with the previous findings of the hydrodynamic-diameter distribution. For the –CH and C–O stretching vibration peaks, the shift was identical for both CUR-SeNP samples, indicating that the interaction between SeNPs and –OH groups also affected the –CH and C–O stretching vibration [23], but the difference in the AN pretreatment intensity did not affect the shift of this peak. The absorption peak around 1606 cm^−1^ was attributed to the adsorbed water blending vibration; the slightly lower wavenumber of CUR-H indicated its higher water solubility, and the addition of SeNPs had no obvious effect on this peak.

Overall, the FTIR results showed that the SeNPs were stabilized through the hydrogen bonding interactions, and the different positions of broad peaks in the spectra of the two CUR-SeNPs samples (3379 cm^−1^ in CUR-SeNPs-L and 3376 cm^−1^ in CUR-SeNPs-H) indicated the different structures of the CUR-SeNPs prepared at different AN pretreatment intensities.

Figure 3b shows the XRD patterns of SeNP control, CUR-L, CUR-H, CUR-SeNPs-L, and CUR-SeNPs-H, respectively. No narrow diffraction peaks were observed in the pattern of SeNPs, indicating that the SeNPs prepared in this work were amorphous. A broad peak around 2q = 20° was observed in all samples containing CUR, which is characteristic of CUR [43]. A low-intensity narrow diffraction peak at around 2q = 28° was observed in the pattern of CUR-L but did not appear in the other patterns. We think this peak was caused by THAs, and its disappearance in the pattern of CUR-SeNPs-L indicated that SeNPs interfered with the re-formation of THAs. In addition, no other narrow diffraction peaks were observed in all samples, indicating that the introduction of CUR did not change the amorphous state of SeNPs.

### 3.3. Surface Micromorphology of CUR-SeNPs

**Figure 4:**
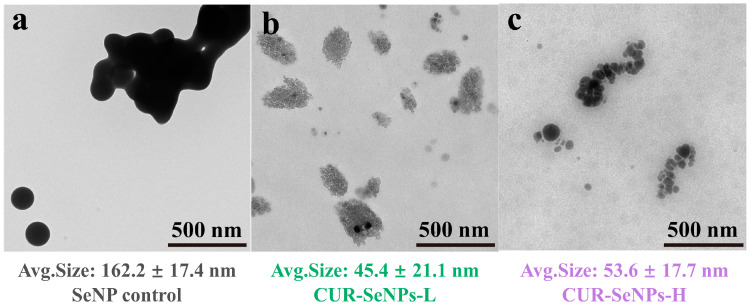
TEM photographs of (**a**) SeNP control, (**b**) CUR-SeNPs-L, and (**c**) CUR-SeNPs-H with their calculated average particle size of SeNPs.

CUR-SeNPs were observed using TEM to more intuitively determine if the SeNPs were encapsulated inside the triple helices. Figure 4a shows the photograph of the SeNP control. The aggregation of SeNPs was serious without the stabilization of CUR, which was obviously not conducive to the stability and storage of SeNPs. The average particle size of the SeNP control was 162.2 ± 17.4 nm, which was similar to the average hydrodynamic diameter of the SeNP control in Table 1. Figure 4b shows the photograph of CUR-SeNPs-L. Many free SeNPs were observed, and many large flake-like substances were believed to be water-insoluble THAs. A few SeNPs were observed inside the THAs, and these SeNPs were believed to have been trapped inside the THAs during the re-aggregation of THAs. The average particle size of individual SeNPs in CUR-SeNPs-L was 45.4 ± 21.1 nm, which was slightly smaller than the average hydrodynamic diameter because of the hydrated layer on the surface of SeNPs.

Figure 4c shows the photograph of CUR-SeNPs-H. THAs were rarely present. The SeNPs showed a worm-like distribution instead of the large agglomerated distribution in the control, and the SeNPs were more clearly divided from one another. Moreover, the SeNPs did not show a very regular spherical feature, which may be caused by squeezing during the rewinding of the triple helix. Therefore, we believe that the SeNPs were encapsulated in the triple helix, and the worm-like structure of triple helix determined the spatial distribution of SeNPs. In addition, as shown in Figure 4, the average particle size of individual SeNPs in CUR-SeNPs-H was 53.6 ± 17.7 nm, which was also slightly smaller than the previous average hydrodynamic diameter, and the ranking of SeNP particle sizes in the three samples was exactly the same as that of *D*_h_ corresponding to the P1 peak in Table 1.

Overall, the particle size of individual SeNPs stabilized by CUR was significantly lower, which was conducive to enhancing the biological activity of SeNPs [24]. Although the particle size of individual SeNPs in CUR-SeNPs-H was slightly larger than that in CUR-SeNPs-L, the former SeNPs were encapsulated inside the triple helix and it did not contain insoluble THAs, which was more favorable for long-term stabilization.

### 3.4. Reactivity of CUR-SeNPs with Reactive Oxygen Species

While different AN pretreatments affected the structure of CUR-SeNPs, the biological activity of SeNPs in both composites was unclear. The physiological activity of SeNPs is usually associated with reactive oxygen species (ROS). Song, Chen, Zhao, Sun, Che, and Leng [44] suggested that the Se^0^↔Se^4+^ cycle is the mechanism of the anti-tumor activity of SeNPs. Therefore, the reactivity of CUR-SeNPs with ROS can reflect the biological activity of SeNPs to some extent. The forms of intracellular ROS in human cells are generally considered to be hydroxyl radicals, superoxide anion radicals, and hydrogen peroxide. Therefore, to better simulate the reaction of CUR-SeNPs with ROS in human cells, the reactivity of CUR-SeNPs with hydroxyl radicals and superoxide anion radicals was determined.

**Figure 5:**
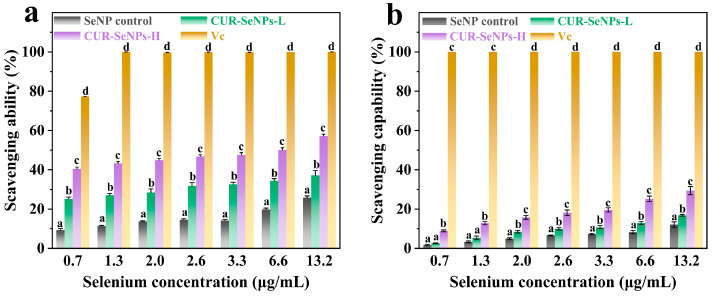
Reactivity of CUR-SeNPs with (**a**) hydroxyl radicals and (**b**) superoxide anion radicals (different letters in the same concentration group indicate significant differences between samples, *p* < 0.05).

The reactivity of CUR-SeNPs with hydroxyl radicals is shown in Figure 5a. The scavenging ability was already about 100% at an ascorbic acid concentration higher than 0.2 mg/mL (Vc concentrations were omitted on the horizontal coordinate in Figure 5), indicating the validity of this hydroxyl-radical-scavenging capacity assay. The scavenging ability of CUR-SeNPs-L and CUR-SeNPs-H for hydroxyl radicals was higher than that of the SeNP control at any selenium concentration. The enhanced scavenging ability of CUR-SeNPs is not due to the reaction of CUR itself with radicals, as CUR has been reported to have very weak antioxidant properties [45]. We believed that the size of SeNPs and the hydrogen bonding interactions between SeNPs and CUR hydroxyl groups were the main reasons for the enhanced scavenging ability. First, there is a negative correlation between the antioxidant activity of SeNPs and their particle size [12], i.e., the smaller the particle size, the stronger the antioxidant activity, because SeNPs with small particle size have a higher specific surface area, which makes them more likely to react with radicals. Secondly, the introduction of hydroxyl groups on the surface of SeNPs can enhance the interaction between CUR-SeNPs and the radicals. For example, the free-radical-scavenging ability of chitosan-stabilized SeNPs was reported to be higher than that of carboxymethyl chitosan-stabilized SeNPs at the same concentration (the particle size difference of SeNPs in the two samples was very small) because there were more C_6_ primary hydroxyl groups converted to carboxylate groups in carboxymethyl chitosan molecules compared to chitosan, which may reduce the total number of hydroxyl groups [46]. In our work, the FTIR results confirmed that more Se···H hydrogen bonds were formed between SeNPs and hydroxyl groups in CUR-SeNPs-H. Although the TEM results and the DLS results showed that the particle size of SeNPs in CUR-SeNPs-H was slightly larger than that of SeNPs in CUR-SeNPs-L, the hydroxyl-radical-scavenging ability of CUR-SeNPs-H was still larger than that of CUR-SeNPs-L, indicating that Se···H hydrogen bond number is more important for the radical-scavenging ability of CUR-SeNPs than the size of SeNPs.

Furthermore, the concentration-scavenging ability of all samples showed a concentration-dependent increase, and the corresponding half-inhibitory concentration (IC_50_) was approximately 6.58 μg/mL (selenium concentration). The reactivity of CUR-SeNPs with superoxide anion radicals is shown in Figure 5b. The pattern between the samples was basically the same as that of the hydroxyl-radical-scavenging ability assay. The main difference was that the reactivity of the CUR-SeNPs with superoxide anion radicals was not as good as that with hydroxyl radicals, similar to the results reported by Zhang, Zhai, Zhao, Ren, and Leng [47]. Overall, the antioxidant activity of CUR-SeNPs-H was better than that of CUR-SeNPs-L.

### 3.5. Storage Stability of CUR-SeNPs

**Figure 6:**
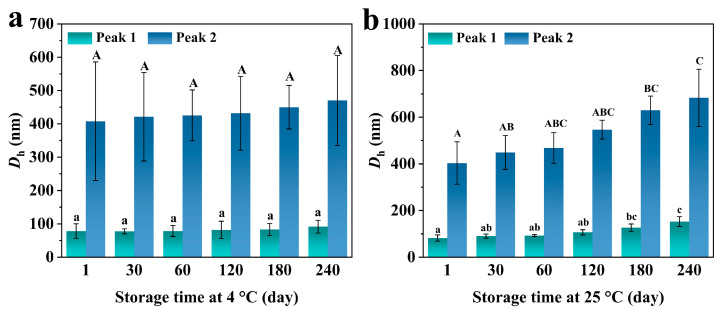
Variation in *D*_h_ of CUR-SeNPs-H with storage time at (**a**) 4 °C and (**b**) 25 °C (different lowercase letters marked on the *D*_h_ of P1 indicate significant differences, *p* < 0.05; different capital letters marked on the *D*_h_ of P1 indicate significant differences, *p* < 0.05).

Figure 6 shows the change in the *D*_h_ of CUR-SeNPs-H with storage time. As described in the previous section, peak P1 and P2 in the *D*_h_ distribution were assigned to free SeNPs and ITHs that contain some SeNPs, respectively. Thus, the changes in their corresponding *D*_h_ are shown separately. At 4 °C (Figure 6a), CUR-SeNPs-H exhibited good storage even at 240 days because no significant changes occurred in the *D*_h_. At 25 °C (Figure 6b), from the first day to 180th day, the *D*_h_ of free SeNPs increased significantly from 81.83 ± 13.39 nm to 126.37 ± 15.86 nm, and the *D*_h_ of combination of SeNPs and triple helix increased significantly from 403.52 ± 90.73 nm to 629.92 ± 60.61 nm. The accelerated aggregation of SeNPs explained these pattern differences at varied temperatures [48]. Although the storage stability of CUR-SeNPs-H at 25 °C was insufficient, it can maintain good stability at 4 °C for 240 days, and to our knowledge, the storage stability of CUR-SeNPs-H is better than that of most other polysaccharide-SeNP composites [17]. As previously described, CUR-SeNPs-H could co-stabilize SeNPs through CUR molecule wrapping and Se···H hydrogen bonding interactions. We believe that this stable triplex wrapping is the main reason that makes CUR-SeNPs-H more stable than other polysaccharide-SeNP composites.

### 3.6. Construction Mechanism of CUR-SeNPs

**Figure 7:**
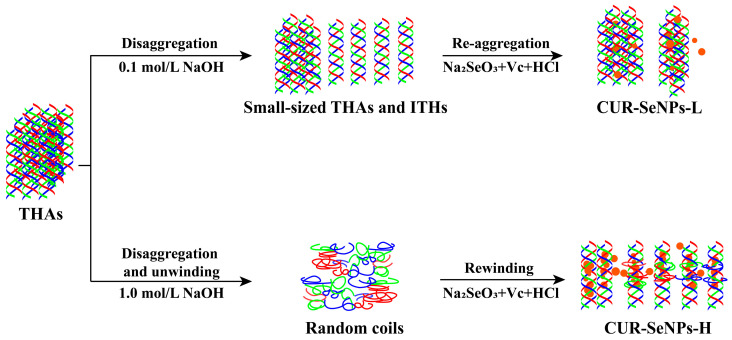
The construction mechanisms of CUR-SeNPs prepared under different AN pretreatments.

Based on all characterization results, the construction mechanisms of CUR-SeNPs under different AN pretreatments were derived. As shown in Figure 7, when the intensity of AN pretreatment was low (*C*_NaOH/HCl_ = 0.1 mol/L), large-sized THAs first disaggregated to small-sized THAs and some ITHs under alkali treatment. After neutralization treatment and initiation of the synthetic reactions of SeNPs, some SeNPs were trapped inside the THAs through the re-aggregation of ITHs while the remaining SeNPs failed to be stabilized and thus dispersed in water. When the intensity of AN pretreatment was high (*C*_NaOH/HCl_ = 1.0 mol/L), THAs first disaggregated and denatured to random coils under alkali treatment. After neutralization and initiation of the synthetic reactions of SeNPs, most SeNPs were stabilized and encapsulated inside the triple helices through the rewinding of ITHs, whereas a few SeNPs were stabilized on the outside of the triple helices or dispersed in water. Obviously, the AN pretreatment intensity determined whether or not SeNPs can be effectively encapsulated inside the triple helix, which was crucial to the stability of SeNPs.

## 4. Conclusions

All results confirmed the hypothesis that different AN pretreatments induced different self-assembly behaviors of CUR, which in turn resulted in prepared CUR-SeNPs with various structures, stabilities, and dispersion properties. At a low AN pretreatment intensity (*C*_NaOH/HCl_ = 0.1 mol/L), only the disaggregations/aggregations of THAs were present, so most SeNPs were free in water and a few SeNPs were trapped in THAs. At a high AN pretreatment intensity (*C*_NaOH/HCl_ = 1.0 mol/L), the ITHs dissociated from THAs undergo further unwinding-rewinding, so most SeNPs were encapsulated inside the cavity during the rewinding of ITHs. From the latter route, SeNPs were better stabilized and dispersed by Se···H hydrogen bonding interactions and physical wrapping of a triple helix, and the CUR-SeNPs had a better antioxidant activity.

Overall, this study constructed the CUR-SeNPs and revealed the mechanism of CUR-SeNPs prepared through different self-assembly behaviors of the triple helix. In addition to providing evidence to the hypothesis initially proposed, this finding can also help broaden the application of CUR and SeNPs. In addition, we believe that the triple-helix self-assembly of CUR will have more potential in nutrition fortification and supplementation research.

In the future, further research and analysis are needed in the following areas, some of which we have already undertaken:

(1) The underlying mechanism of the Se···H hydrogen bonding interaction between SeNPs and CUR (or other polysaccharides);
(2) The maximum loading of SeNPs in CUR helices and methods for their detection;
(3) Further investigation of other biological activities of CUR-SeNPs, such as anti-tumor activity, immunomodulatory activity, etc.

## Figures and Tables

**Table 1:** Hydrodynamic-diameter distributions of CUR-SeNP samples.

Sample Name	SeNP Control	CUR-L	CUR-H	CUR-SeNPs-L	CUR-SeNPs-H
z-average (nm)	159.1	238.7	375.1	230.1	280.6
PDI	0.013	0.563	0.022	0.464	0.302
*D*_h-P1_ (nm) by intensity	164.70 ± 36.04	/	/	61.08 ± 15.89	78.24 ± 21.84
*D*_h-P2_ (nm) by intensity	/	280.7 ± 168.3	381.3 ± 24.93	321.90 ± 144.40	407.80 ± 178.00
*D*_h-P3_ (nm) by intensity	/	5231 ± 456.8	/	4737.00 ± 760.00	/
*D*_h-P1_ (nm) by volume	163.00 ± 44.34	/	/	50.67 ± 15.07	63.25 ± 17.43
*D*_h-P2_ (nm) by volume	/	421.6 ± 236.3	378.3 ± 47.51	433.20 ± 151.90	503.60 ± 171.50
*D*_h-P3_ (nm) by volume	/	5331 ± 694.2	/	4978.00 ± 861.30	/

**Table 2:** The differences in the main absorption peaks of stretching vibrations between samples.

Sample Name	CUR-L	CUR-H	CUR-SeNPs-L	CUR-SeNPs-H
Stretching vibration of –OH	3385 cm^−1^	3385 cm^−1^	3379 cm^−1^	3376 cm^−1^
Stretching vibration of C–H	2881 cm^−1^	2880 cm^−1^	2891 cm^−1^	2891 cm^−1^
The bond between polysaccharide molecule and water	1606 cm^−1^	1604 cm^−1^	1605 cm^−1^	1604 cm^−1^
Stretching vibration of C–O	1029 cm^−1^	1029 cm^−1^	1039 cm^−1^	1039 cm^−1^
β-glycosidic bond	890 cm^−1^	890 cm^−1^	890 cm^−1^	890 cm^−1^

## Data Availability

Data are contained within the article.

## Supplementary Materials

The original graphs of the hydrodynamic-diameter distributions and zeta potentials in this paper are available for download at https://www.mdpi.com/article/10.3390/foods12030563/s1: Figure S1: Original graphs of the (a) hydrodynamic-diameter distribution by intensity, and (b) hydrodynamic-diameter distribution by volume of the CUR-SeNPs samples. Figure S2: Original graphs of the zeta potentials of the CUR-SeNPs samples.
