# Supplementary material for: Constructing Selenium Nanoparticles with Enhanced Storage Stability and Antioxidant Activities via Conformational Transition of Curdlan
Source: Foods. 2023 Jan 27;12(3):563. doi: 10.3390/foods12030563 (PMC9914686; doi:10.3390/foods12030563)
Supplement: Supplementary file 1 [file foods-12-00563-s001.zip › foods-2149163-supplementary.pdf]

Supplemental Files

Article Title: Constructing selenium nanoparticles with enhanced storage stability and antioxidant activities via conformational transition of curdlan

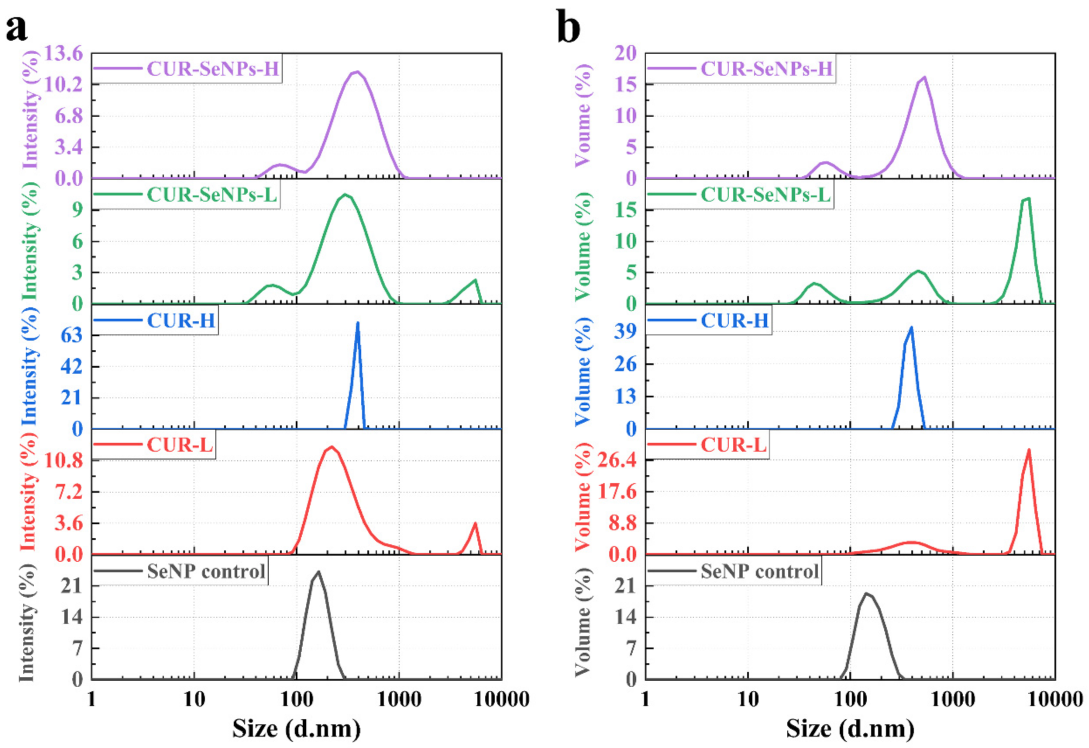

Figure S1. Original graphs of the (**a**) hydrodynamic-diameter distribution by intensity, and (**b**) hydrodynamic-diameter distribution by volume of the CUR-SeNPs samples.

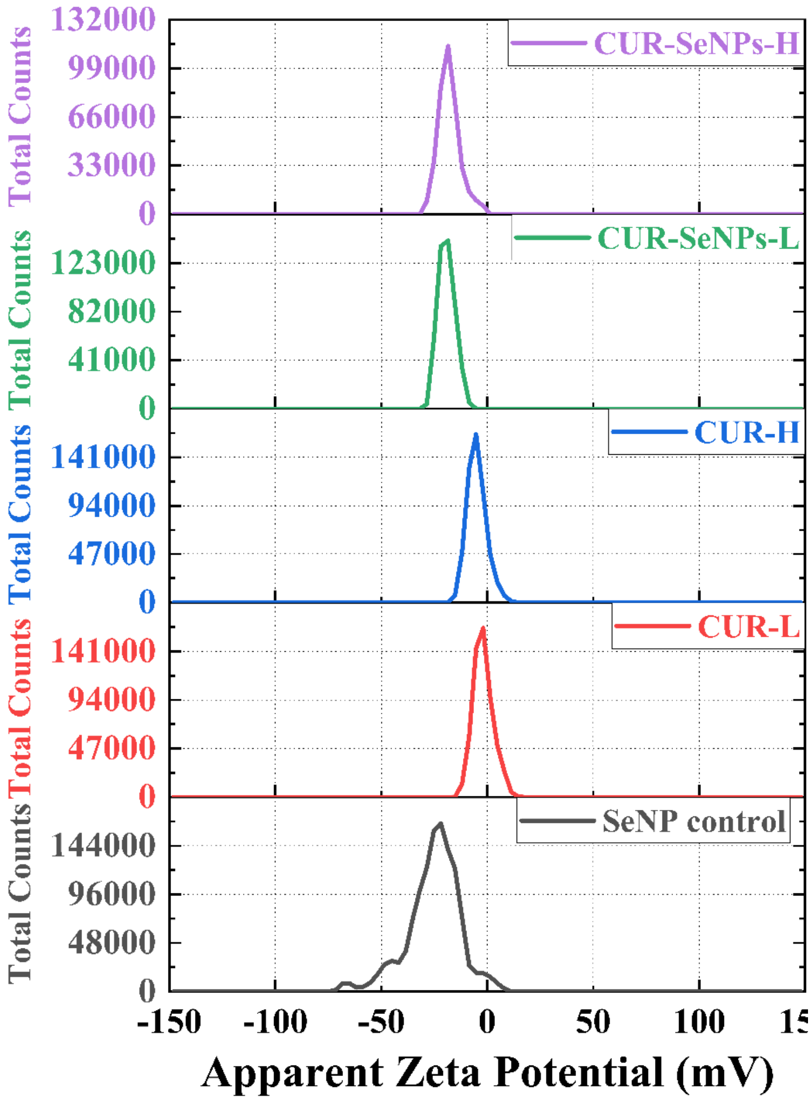

Figure S2. Original graphs of the zeta potentials of the CUR-SeNPs samples.
